# The structural model of cyberchondria based on personality traits, health-related metacognition, cognitive bias, and emotion dysregulation

**DOI:** 10.3389/fpsyt.2022.960055

**Published:** 2023-01-09

**Authors:** Mohammad Nasiri, Shahram Mohammadkhani, Mehdi Akbari, Majid Mahmoud Alilou

**Affiliations:** ^1^Department of Clinical Psychology, Faculty of Psychology and Education, Kharazmi University, Tehran, Iran; ^2^Department of Psychology, Faculty of Psychology and Educational Sciences, University of Tabriz, Tabriz, Iran

**Keywords:** cyberchondria, cognitive bias, health-related metacognition, emotion dysregulation, personality traits

## Abstract

**Introduction:**

Cyberchondria is excessive seeking for online health-related information related to increasing health anxiety and distress levels. The current study investigated the mediating role of health-related metacognition, cognitive bias, and emotion dysregulation in the relationship between personality traits and cyberchondria.

**Methods:**

Participants were 703 individuals 18+ years old who had access to the internet (males = 43.8%, mean age = 33.82 ± 10.09 years and females = 56.2%, mean age = 34.37 ± 11.16 years). They voluntarily completed a questionnaire package that included the Cyberchondria Severity Scale (CSS), the revised NEO Personality Inventory (NEO-PI-R), the Difficulties in Emotion Regulation Scale (DERS), the Meta-Cognitions about Health Questionnaire (MCQ-HA), and the Health Cognitions Questionnaire (HCQ).

**Results:**

The initial evaluation of the model demonstrated that the personality traits of openness to experience, agreeableness, and conscientiousness had no significant relationship with other variables in the structural model, and the effects of neuroticism and extroversion were the only significant results. Rerunning the model with the removal of non-significant variables revealed a full mediation of health-related metacognition, cognitive bias, and emotion dysregulation in the relation between personality traits (neuroticism and extraversion) and cyberchondria. Fit indices demonstrated the acceptable fit of the model with the collected data (χ^2^ = 979.24, *p* <.001; NFI = 0.92, CFI = 0.93, GFI = 0.90, IFI = 0.93, RMSEA = 0.071, and SRMR = 0.063). The results indicated that the present model could explain *R*^2^ = 54% of cyberchondria variance.

**Discussion:**

These findings suggest that health-related metacognition, cognitive bias, and emotion dysregulation could demonstrate a full mediating role in the correlation between personality traits and cyberchondria.

## 1. Introduction

Increasing advances in medical, psychology, and physiological studies have led to novel viewpoints regarding health and disease. In this regard, according to the bio-psycho-social approach, disease and health could be assigned as the products of a combination of biological features (e.g., genetics), behavioral characteristics (e.g., stress, lifestyle, health beliefs), and cultural situations (e.g., family relationships, cultural effects, and social support) (1). The role of psychology has been highlighted in the conceptualization of health and disease, particularly in medical problems and health care (2). Research demonstrated that a significant percentage of complaints and physical symptoms patients report to physicians have no medical justification and are better explained by psychological processes (3, 4). Therefore, defining the problematic patterns associated with physical symptoms can be among the top research areas in health psychology.

The aforementioned problematic patterns associated with physical symptoms revealed various aspects of the age of technology and information. The growing popularity of the internet has made easy-to-access health information feasible for the public. With the advent of free and straightforward access to health-related information online, searching the web has become the prevalent method of obtaining medical and health information (5, 6). Online access to medical and health-related information could demonstrate a beneficial potential, such as enhancing people’s awareness of the nature, causes, and prevention/treatment methods of various diseases. However, the internet is frequently utilized as a source of self-diagnosis and reassurance for individuals concerning their health (7, 8). Individuals with severe anxieties regarding their health devote extra time searching for health information (9), and they regularly refer to the internet to obtain information (10).

Anxiety associated with searching for health information online once it is frequent and extreme is called cyberchondria (11, 12). In other words, cyberchondria refers to the extreme search for information regarding medical care and health on the Internet (13). Cyberchondria has been widely defined as the recurrent and anxiety-driven online search for health information that can amplify the existing anxiety or distress about the health condition (11).

It should be noted that the distinction between the online search for health information and cyberchondria is related to its behavioral results (11). This means that cyberchondria denotes an online search for health information and comprises the extreme and repetitive search that is driven by or led to anxiety. Therefore, surfing the internet in order to seek information related to health is not maladaptive in its sense, and it becomes a pathological pattern once it is extreme and accompanied by intensified anxiety. The critical point is that cyberchondria is an abnormal pattern of behavior and not a distinct diagnostic category (14). However, it is abundant among people who have demonstrated higher levels of health anxiety.

A critical factor in cyberchondria is the ambiguity regarding available health-related information available on the internet, which is regularly disorganized, incomplete, and misleading (15, 16). Accordingly, individuals in quest of reassurance about their health devote plenty of time to check the accuracy of online information. This process leads to a cycle in which the extreme search for information surges stress and anxiety (11).

Although the definition and conceptualization of cyberchondria are diverse in theoretical and research background, two variables, including behavioral and emotional components, can be considered over which researchers have a decent sum of consensuses. The behavioral component of cyberchondria contains the extreme and frequent Internet exploration for acquiring health-related information comparable to reassurance behavior. This behavior primarily reduces anxiety and fear of illness or uncertainty regarding physical symptoms. Although the abovementioned search occasionally reduces anxiety, excessive preoccupation with health and physical symptoms leads to a long-term pathological pattern. The emotional component of cyberchondria also encompasses distress and anxiety caused by search behavior or the inability to control search behavior (8).

Cyberchondria, on the other hand, empirically consists of four dimensions: (1) compulsion, i.e., the degree to which Internet search for health information interferes with daily activities; (2) distress, i.e., the tendency to experience anxiety while searching the internet; (3) excessiveness, i.e., the repetitive quality of the individual search, and (4) reassurance, i.e., the degree of the individual’s tendency to receive medical advice (17, 18).

A review of the research background in the field of cyberchondria indicated that considerable efforts have been spared to identify the factors influencing the development of this phenomenon [e.g., (19–26)]. However, practically there has been no available study to comprehensively assess the factors affecting the development and persistence of cyberchondria. Previous analyses merely considered similar structures such as illness anxiety disorder, health anxiety, and somatic symptom disorder. Thus, the gap is felt in the body of literature to assess the main predictors and factors influencing cyberchondria and how they interact. Therefore, the present study aimed to evaluate the structural model of cyberchondria based on personality traits, health-related metacognition, cognitive bias, and emotion dysregulation.

A structural model was used in the present study to clarify the etiology of the cyberchondria phenomenon and explain its predictor variables in the form of a coherent framework. In this model, it is assumed that in addition to having a direct effect on cyberchondria, personality factors demonstrate an indirect effect on cyberchondria through the mediation of health-related cognitive bias, health-related metacognitions, and emotional dysregulation. In the following, an attempt will be made to review the research support for each component of the proposed structure. Considering that the research background in the field of cyberchondria is exceedingly new, studies in health anxiety have also been utilized to support the hypothetical model of the present study.

### 1.1. Personality traits: Underlying factor in cyberchondria

One of the main factors in the present research model is personality traits. Some studies considered personality tendencies to experience various emotions as an essential factor in explaining interpersonal dissimilarities in the process of health anxiety experience and the search for health information (27–29). Previous findings have suggested that the five-factor model of personality and various psychopathologies are related (30). Low extraversion, high neuroticism, and low conscientiousness are significantly associated with anxiety disorders, such as health anxiety (31, 32). The personality traits are also demonstrated to be correlated with behaviors related to health (33), medical and health information-seeking behaviors (34–36), and Health problems caused by internet use (37, 38). Also, a series of studies showed that cyberchondria is correlated explicitly with neuroticism (39, 40) and negative emotions (20, 22, 39).

### 1.2. Health-related cognitive bias in the relationship between personality traits and cyberchondria

Mediating variables in the current research model include health-related cognitive bias, health-related metacognitions, and emotional dysregulation. Explaining cyberchondria based on cognitive components such as health-related cognitive bias can be a fundamental step in the etiology of this phenomenon. Significant empirical evidence suggests that individuals with health anxiety and concerns have maladaptive beliefs regarding the disease and its symptoms (41–43). The central theme of these beliefs revolves around the severity and perceived probability of a person developing the disease (41) and considering the unexpected symptoms as a severe disease (44).

Based on cognitive theories, it can be argued that health anxiety, somatic symptoms, and medical information regarding the disease might be perceived as over-threatening than what they are. Therefore, the perceived probability of illness is higher than its actual probability. Exposure to information regarding the signs and symptoms of the disease can also lead to catastrophizing, misunderstanding, and negative thoughts (42, 45, 46). Thus, the catastrophizing of the pain and physical symptoms can exacerbate health anxiety and, in turn, lead to an extreme search for health information on the Internet (23).

Anxiety and health concerns are formed in cognitive-behavioral models because of misinterpretations of emotions and bodily symptoms (47). Abramowitz and Braddock (47) showed that concerns regarding health upsurges physiological arousal, which leads to increased levels of emotion and physical symptoms, causing the person to interpret harmless signs and symptoms as threatening. Clark (48) also considered that the individual’s belief in their health being in danger leads to hypervigilance and extreme attention to the signs of the threat, as a result of which constant vigilance and frequent search for symptoms are required. Various health anxiety-related disturbing and unwanted thoughts focus on the past and future (49). Individuals with cyberchondria also seem to have an unpleasant preoccupation with their health and overestimate the possibility of the disease’s development. They also overestimate the risk of the disease. Therefore, they develop in-mind catastrophe and consider their disease highly disabling and deadly. Changes in physical senses, function, and physical appearance lead to disturbing thoughts and cognitive biases in these individuals. They also overestimate their vulnerability to disease and underestimate their resilience (49).

These maladaptive health cognitions may lead to pathological behaviors such as cyberchondria under personality traits. Among personality traits, a plethora of research evidence has been obtained for the role of neuroticism and extraversion in dysfunctional cognitive processes. Considering that neuroticism is associated with high psychological distress and maladaptive coping strategies (50), individuals with high neuroticism develop more negative beliefs regarding their physical health (51). Contrasting to people with high neuroticism, individuals with high levels of extraversion develop more positive health-related expectations and beliefs (52). Therefore, the interaction and interactive effects between these personality traits and cognitive biases can lead to cyberchondria-related behaviors.

### 1.3. Health-related metacognitions in the relationship between personality traits and cyberchondria

Another mediating variable in the present research model is health-related metacognitions. The metacognitive theory believes metacognition can be more significant than cognition (53). According to the theory of metacognition, a person’s beliefs regarding the uncontrollability and dangerousness of some thoughts can predict psychological disorders (54) and health anxiety, particularly (55, 56). Bailey and Wells (56) showed that metacognitive beliefs, such as negative beliefs regarding uncontrollability and anxiety risk, intensify individuals’ sensitivity to physical signs and symptoms. Kaur et al. (55) reported that metacognition is positively associated with a bias toward positive and negative health information. Barenbrügge et al. (57) also found that metacognition relates to various aspects of health anxiety, such as belief about illness, physical complaints, and multiple medical consultations. Various studies have also considered the interaction of metacognition with other factors in the formation of worry and anxiety concerning physical symptoms. In a study by Bailey and Wells (58), they found that metacognition is related to health anxiety and moderates the relationship between catastrophizing and health anxiety. Melli et al. (59) also showed that the metacognitive belief of uncontrollability and thoughts regarding disease interference could predict health anxiety and modulate the relationship between anxiety sensitivity and health anxiety.

The interaction of personality factors with health-related metacognitions is an essential part of the conceptual model of the present study. In this context, neuroticism is more prominent among personality traits. Studies suggest that neuroticism, as a temperamental underlying, can activate maladaptive metacognitive responses (60). Spada et al. (54) showed that anxiety-oriented metacognitive beliefs and pain catastrophizing could mediate the relationship between neurotic personality traits and pain behavior. Another study disclosed that metacognitive beliefs mediate the effect of neuroticism on dysfunctional behaviors and psychological distress (61, 62).

### 1.4. Emotional dysregulation in the relationship between personality traits and cyberchondria

The third mediating variable of the hypothetical cyberchondria model in the present study is emotion dysregulation. Emotion regulation can be considered conscious and unconscious strategies to increase, decrease, and maintain emotional responses (63). Gross and Jazaieri (64) distinguished three critical factors in regulating maladaptive emotions. (1) Awareness and understanding of the emotional context can be incompatible in both high and low consciousness. (2) Problem-based emotion regulation goals (e.g., someone who believes their health is in danger and is therefore highly concerned and engages in safety behaviors) and (3) Problem-solving emotion strategies that lead to the formation of dysfunctional and traumatic cognitions.

Emotional dysregulation is influenced by personality factors and can affect health anxiety and cyberchondria. In a meta-analysis conducted by Barańczuk (65), it was shown that neuroticism has a positive relationship with avoidance and rumination strategies and a negative association with mindfulness and re-evaluation strategies. Besides, Extraversion has a positive relationship with re-evaluation, acceptance, and problem-solving strategies and a negative relationship with suppression, avoidance, and rumination strategies. On the other hand, the components mentioned above, along with difficulty in identifying and expressing emotions on health anxiety, have frequently been cited in the context of health anxiety and physical disorders (66, 67). Studies by Bardeen and Fergus (68) reported that emotion suppression, difficulty in devising control over the impulse, and having restrained access to efficient emotion regulation-related strategies are the most crucial contributors to health anxiety. Findings that examined the cognitive, behavioral, and emotional dimensions of health anxiety suggested that the behavioral dimension of health anxiety, i.e., reassurance, is used as an alternative strategy for the lack of adaptive emotion regulation strategies (66, 69).

**FIGURE 1:**
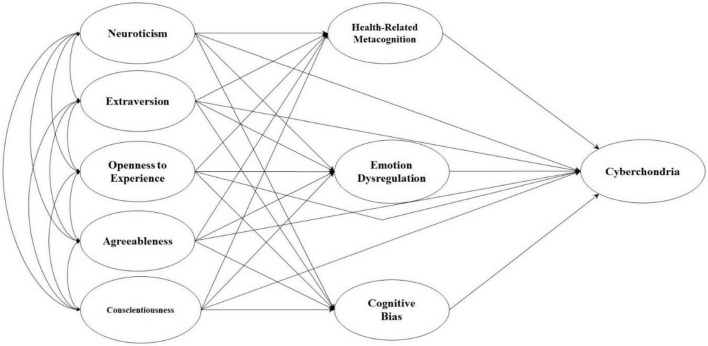
Conceptual model of cyberchondria.

In short, cyberchondria can be predicted based on personality traits and maladaptive cognitive, metacognitive, and emotional dysregulation. The relationship between the variables of the present research seems moderately clear. The area that requires further discussion and analysis is how the abovementioned variables are placed in an organized model framework to elucidate the relationships. Therefore, the present study aimed to test the structural model of cyberchondria based on personality traits, metacognition, and cognitive biases related to health and emotional dysregulation. In order to test the present model, a mediation model format was utilized in which two sets of vulnerabilities interact with one another, eventually leading to cyberchondria. Figure 1 illustrates the hypothetical model of the present research.

## 2. Materials and methods

### 2.1. Procedure and participants

The present study was designed to be of a descriptive correlational design with its statistical population comprising all individuals older than 18 years who had access to the internet and not suffering from a severe illness. Using the availability sampling method, 750 individuals participated in the present study, which plunged to 703 after eliminating outliers. Therefore, the final data analysis contained 703 participants. The study sample comprised 395 females (56.2%) and 308 males (43.8%). The mean age and standard deviation of the men’s age were 33.82 and 10.09, respectively, and the mean age and standard deviation of the women’s age were 34.37 and 11.16, respectively. In terms of education, 28 participants (4%) held a post-diploma degree, 136 (19.3%) held a diploma, 208 (29.6%) held a bachelor’s degree, 268 (38.1%) held a master’s degree, and 63 (9%) held a Ph.D. Furthermore, in terms of marital status, 381 (54.2%) were married, 301 (42.8%) were single, and 21 (3%) were divorced.

The participants were involved in the study via invitations through social media and received the necessary information regarding the research. The study was conducted on the Iranian population from June 2021 to December 2021. The participants responded to the online questionnaires and sent their answers to the researcher. Inclusion criteria included: being over 18 years of age, having access to the internet, being willing to participate in the study, approval of written informed consent, and not suffering from a severe physical or mental illness (Severe physical illness including infectious disease, cardiovascular disease, gastrointestinal disorder, endocrine disorder, urinary disease, malignant tumors, disability, and substance abuse problems. Severe mental illness, including schizophrenia, bipolar and depressive disorders, and their related spectrum disorders). Participants completed research questionnaires that included the Cyberchondria Severity Scale (CSS), The revised NEO Personality Inventory (NEO-PI-R), the Difficulties in Emotion Regulation Scale (DERS), The Meta-Cognitions about Health Questionnaire (MCQ-HA), and The Health Cognitions Questionnaire (HCQ).

### 2.2. Measures

#### 2.2.1. Cyberchondria severity scale

The CSS was developed by McElroy and Shevlin (70) and contained 33 questions assessing the anxiety and distress associated with the online exploration of health-related information. Scale questions are scored on a 5-point Likert scale (1 = never, 2 = rarely, 3 = sometimes, 4 = often, 5 = always). The scale has a total score as well as 5 sub-scales: (A) compulsion (questions: 8, 3, 25, 17, 24, 12, 14, 6), (B) Distress (questions: 5, 29, 31, 22, 10 20, 7, 23), (C) Excessiveness (questions: 13, 18, 2, 19, 30, 11, 1, 21), (D) Reassurance (questions: 15, 27, 4, 26, 32, 16), and (E) Mistrust of Medical Professionals (questions: 9, 28, 33). The course is scored in reverse with questions 9, 28, and 33. The present scale was undergone the standardization process in Iran by Nasiri et al. (18), during which the Mistrust of Medical Professionals factor was eliminated due to having a factor load of less than 0.30, and the factors of compulsion, Distress, Excessiveness, Reassurance were approved. Moreover, in Nasiri et al. (18), the scale’s reliability was reported using Cronbach’s alpha test equal to α = 0.91. In addition, the subscales of the present questionnaire exhibited good internal consistency (0.62–0.87).

#### 2.2.2. The revised NEO personality inventory

Personality traits were measured in the present study using a five-factor NEO personality questionnaire containing 60 questions (71). The instrument measures 5 subscales of neuroticism, extraversion, openness to experience, agreeableness, and conscientiousness. Each subscale consists of 12 questions scored on a 5-point Likert continuum (1 = strongly disagree to 5 = strongly agree). Cronbach’s alpha of the factors indicated a high degree of internal consistency (0.86–0.92), and its differential and convergent validity have also been confirmed (71). The validity and reliability of the Persian version of the questionnaire have also been confirmed (72).

#### 2.2.3. Difficulties in emotion regulation scale

Brief Version of the DERS is a self-report index designed to assess difficulties in emotion regulation (73). The original form of DERS was created by Gratz and Roemer in 2004 and contained 36 terms (74). The short form of this scale (16DERS-) has 16 items, and the answers are graded on a 5-point Likert scale (1 = almost never to 5 = almost always). The Brief Version of the Difficulties in Emotion Regulation Scale (DERS-16) was designed to measure five of the six factors measured by the original form (DERS). These five factors incorporate: (1) non-acceptance of negative emotions (9, 10, 13), (2) inability to engage in goal-directed behaviors over the distressed periods (3, 7, 15), (3) difficulties in controlling impulsive behaviors over the distressed periods (4, 8, 11), (4) limited access to emotion regulation strategies perceived as practical (5, 6, 12, 14, 16), (5) lack of the emotional clarity (1, 2). This questionnaire has 5 subscales, and a total score is obtained from the sum of all 5 subscales. In addition to saving time in terms of convergence and differential validity, the study of Bjureberg et al. revealed that DERS-16 is comparable to the long 36-item DERS form and retains a decent internal consistency (α = 0.92). Besides, its test-retest reliability (ρI = 0.85 and *P* < 0.001) was appropriate (73). Shahabi et al. (75) validated the present scale in Iran. Their study results indicated that the Persian version of this scale held a good internal consistency (α = 0.91) along with a decent test-retest reliability (ρI = 0.92 and *P* < 0.001) and concurrent validity (75).

#### 2.2.4. The meta-cognitions about health questionnaire

The MCQ-HA was developed by Bailey and Wells (76), and it is on the basis of the extensively utilized General Metacognitive Belief Scale (MCQ) (77). Nevertheless, the Metacognitive Beliefs Questionnaire about health has been developed to measure metacognitive beliefs related to health anxiety. This scale consists of 14 questions, and grading the answers is based on the 4-point Likert scale (1 = disagree to 4 = strongly agree). Initial exploratory analysis (76) obtained a three-factor structure consisting of the following subscales: (1) the belief that Thoughts could cause illness (MCQ-HAC); for instance, “Thinking negatively can increase my chances of disease.” (2) The Beliefs about Biased Thinking (MCQ-HAB); for example, “I will be punished for thinking I am in good health.” (3) The belief that Thoughts are Uncontrollable (MCQ-HAU); for example, “I have no control over thinking about my health.” This three-factor structure was also confirmed through CFA. The questionnaire showed good reliability and predictive, convergent, and differential validity.

#### 2.2.5. The health cognitions questionnaire

The HCQ was developed by Hadjistavropoulos et al. (78). The questionnaire consists of 20 questions that measure dysfunctional beliefs related to health anxiety based on the cognitive conceptualization of health anxiety by Salkovskis and Warwick (79). The questionnaire is graded on a 5-point Likert scale (1 = strongly disagree to 2 = strongly agree). HCQ entails 4 subscales: (1) Likelihood of contracting or having an illness (HCQ-L), (2) Awfulness of illness (HCQ-A), (3) Inability to cope with illness (HCQ-C), and (4) Inadequacy of medical services for treating illness (HCQ-M). This questionnaire comprises two forms: one for individuals diagnosed with an illness and the other for those not diagnosed with any disease. In the present study, the second form was utilized. It should be noted that fitting predictive and differential validity have been observed for the current questionnaire with high internal consistency (78). Cronbach’s alpha for the current study sample: HCQ-L: 0.86; HCQ-A: 0.87; HCQ-C: 0.87; HCQ-M: 0.70; total: 0.85.

### 2.3. Statistical analyses

Initially, preliminary analyses of research variables, including descriptive indicators (mean, standard deviation, skewness, and kurtosis), and data screening were performed. Afterward, correlation methods, exploratory factor analysis (EFA), confirmatory factor analysis (CFA), and internal consistency evaluation were used in the inferential analyses.

Later, a two-step SEM approach proposed by Anderson and Gerbing (80) was utilized. In the first step, the measurement model was evaluated using CFA. Subsequently, the structural model presented in the current study was assessed using structural equation modeling (SEM). The model was tested using the MLE method, and the model’s fitness was evaluated according to the fit indices. The following criteria were frequently used to check the fit of a model: Chi-square index (χ^2^), χ^2^/df (acceptable fit ≤ 5(, comparative fit index (CFI) (CFI; acceptable fit ≥ 0.90), Goodness of Fit Index (GFI) (GFI; acceptable fit ≥ 0.90), Incremental Fit Index (IFI) (IFI; acceptable fit ≥ 0.90), Normed fit index (NFI) (NFI; acceptable fit ≥ 0.90), Root Mean Square Error of Approximation (RMSEA) (RMSEA; acceptable fit ≤ 0.06), and Standard Root Mean Square Residual (SRMR) (SRMR; acceptable fit ≤ 0.08) (81).

Before performing SEM analysis, appropriate indicators should be selected for latent variables of research. In the present study, using CFA (measurement model), the power of indicators to measure latent underlying variables was evaluated. For personality traits (neuroticism, extraversion, openness to experience, agreeableness, and conscientiousness variables), considering that they had 12 questions and lacked subscales, EFA (82) was performed to select the indicator variables by fixing on 3 factors and with varimax rotation.

Additionally, subscales of health-related metacognitions (HAB, HAC, HAU), subscales of difficulty in emotion regulation (non-acceptance of negative emotions, inability to engage in goal-directed behaviors over the distressed periods, difficulties in controlling impulsive behaviors over the distressed periods, limited access to emotion regulation strategies perceived as practical, lack of the emotional clarity), subscales of cognitive bias on health (Likelihood of contracting or having an illness, Awfulness of illness, Inability to cope with illness, Inadequacy of medical services for treating illness), and Cyberchondria subscales (Compulsion, Distress, Excessiveness, Reassurance) were selected as indicators of latent variables and were assessed by CFA. However, because the Inadequacy of medical services for treating illness subscale had a factor load of less than 0.30, it was eliminated from the final analysis. For the analysis of the mediation effect, the bootstrapping method to estimate the indirect effect was carried out, and 95% confidence intervals were estimated (83). The number of bootstrap samples was 5,000. Data analysis of the present study was performed using SPSS-25 software along with LISREL 8.80 (84).

## 3. Results

### 3.1. Structural equation modeling assumptions

Before evaluating the structural model of the research, the underlying assumptions of SEM were examined. The realization of these assumptions confirmed the appropriateness of using this statistical method for the present study. One of the critical assumptions of this statistical approach is univariate and multivariate normality. The skewness and kurtosis of the observed variables are employed to check the univariate normality. The skewness of the variables is in the range of −0.088 to 1.437, and their kurtosis is in the range of −0.824 to 2.048. Chou and Bentler (85) considered the cut-off point of ± 3 to be the appropriate range for skewness. For the kurtosis point, values greater than ± 10 are considered problematic for this index (86). The Relative Multivariate Kurtosis Index, calculated to evaluate the assumption of multivariate normality, was 1.281. Bentler (87) supposed that multivariate normality is achieved once the value of this index is not more than 3. The correlation matrix between the observed variables could show the existence of multiple collinearities, with correlation coefficients above 0.85, making it challenging to estimate the model accurately (86). Correlation coefficients in the present study were in the range of −0.424 to 0.783. Univariate outliers were identified using box plots (Outliers < 1st Quartile – 1.5* Interquartile range, outliers > 3rd Quartile + 1.5* Interquartile range), and multivariate outliers were identified using Mahalanobis distance [The cut-off for eight predictive variables and the α value of 0.001 suggested by Tabachnick and Fidell (88) is 26.12]. Preliminary examinations showed that the data were suitable for using the SEM method and the maximum likelihood estimation method (MLE).

Table 1 displays the correlation matrix between the latent variables of the research, along with their mean, standard deviation, skewness, and kurtosis. As presented in the table below, the correlation between the pairs of variables was in the range of −0.466 to 0.523. The results showed that neuroticism, extraversion, and agreeableness were significantly correlated with mediating variables (MCQ-HA, DERS, and CHQ). In addition, all mediator variables demonstrated a significant relationship with cyberchondria.

**TABLE 1:** Mean, standard division, skewness, kurtosis, and correlation matrix of research variables (*N* = 703).

Variables	Mean	SD	Skew	Kurt	1	2	3	4	5	6	7	8
1. NEURO	33.39	8.68	0.159	-0.55	1							
2. EXTRA	37.94	9.2	-0.156	-0.918	-0.466**	1						
3. OPEN	40.69	6.38	-0.217	-0.18	-0.157**	0.152**	1					
4. AGREE	39.66	7.99	-0.259	-0.575	-0.143**	0.193**	0.117*	1				
5. CONS	44.02	7.56	-0.619	0.013	-0.121*	0.101*	0.150**	0.198**	1			
6. MCQ-HA	26.73	6.38	0.385	-0.621	0.329**	-0.333**	-0.144**	-0.105*	-0.097*	1		
7. DERS	35.74	11.05	0.855	0.237	0.514**	-0.402**	-0.093	-0.144**	-0.072	0.523**	1	
8. HCQ	44.67	9.28	0.645	0.356	0.477**	-0.417**	-0.066	-0.145**	-0.110*	0.339**	0.437**	1
9. Cyber	68.91	17.86	0.247	-0.18	0.392**	-0.378**	-0.118*	-0.142**	-0.154**	0.462**	0.480**	0.405**

SD, standard division; Skew, Skewness; Kurt, kurtosis; NEURO, Neuroticism; EXTRA, Extraversion; OPEN, openness to experience; AGREE, agreeableness; CONS, conscientiousness; MCQ-HA, the meta-cognitions about health questionnaire; DERS, difficulties in emotion regulation scale; HCQ, The health cognitions questionnaire; **p < 0.01; *p < 0.05.

### 3.2. Measurement model

The fit indices of the measurement model (CFA) indicated the acceptable fit of the present model [Chi-square index (χ^2^) = 1864.31, χ^2^/df = 4.43, GFI = 0.90, CFI = 0.93, IFI = 0.93, NFI = 0.92, RMSEA = 0.071, SRMR = 0.063]. The selected indicators for each variable are reported in Table 2.

**TABLE 2:** Standard coefficients, Non-standard coefficients, and *T*-values of the observable variables in the measurement model.

Variables	Non-standard coefficients	Standard coefficients	*T*-value
**Neuroticism**			
Neuro 1	2.44	0.76	21.31
Neuro 2	2.20	0.69	19.02
Neuro 2	3.06	0.77	21.76
**Extraversion**			
Extra 1	3.18	0.82	23.96
Extra 2	2.10	0.62	17.04
Extra 3	3.02	0.84	24.72
**Openness to experience**			
Open 1	1.81	0.56	11.35
Open 2	1.84	0.65	12.48
Open 3	1.26	0.49	10.24
**Agreeableness**			
Agree 1	1.48	0.48	11.98
Agree 2	2.99	0.84	19.70
Agree 3	2.45	0.74	17.72
**Conscientiousness**			
Cons 1	2.26	0.72	16.37
Cons 2	2.16	0.63	14.78
Cons 3	1.86	0.61	14.21
**The meta-cognitions about health questionnaire**			
MCQ-HAB	1.34	0.59	15.93
MCQ-HAC	1.82	0.59	15.70
MCQ-HAU	2.16	0.88	25.49
**Difficulties in Emotion Regulation Scale**			
Clarity	0.78	0.57	15.81
Strategies	3.45	0.91	30.26
Impulse	2.11	0.80	24.70
Goals	1.87	0.71	21.19
Non-acceptance	2.29	0.81	25.53
**The health cognitions questionnaire**			
Difficulty coping	3.73	0.74	20.69
Likelihood of illness	1.90	0.64	17.08
Awfulness of illness	2.46	0.78	22.08
**Cyberchondria**			
Compulsion	3.62	0.72	21.25
Distress	3.05	0.69	19.93
Excessiveness	5.95	0.92	29.80
Reassurance	3.57	0.64	18.06

Clarity, lack of the emotional clarity; Strategies, limited access to emotion regulation strategies perceived as effective; Impulse, difficulties in controlling impulsive behaviors over the distressed periods; Goals, inability to engage in goal-directed behaviors over the distressed periods; Non-acceptance, non-acceptance of negative emotions; Neuro 1, Neuro 2, Neuro 3, Parcels of Neuroticism; Extra 1, Extra 2, Extra 3, Parcels of Extraversion; Open 1, Open 2, Open 3, Parcels of Openness to experience; Agree 1, Agree 2, Agree 3, Agreeableness; Cons 1, Cons 2, Cons 3, Conscientiousness.

### 3.3. Structural model

**FIGURE 2:**
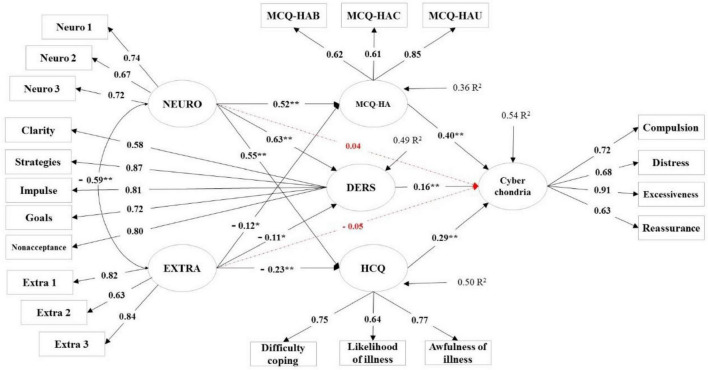
Structural equation modeling of cyberchondria with standard coefficients. NEURO, neuroticism; EXTRA, extraversion; MCQ-HA, The Meta-Cognitions about Health Questionnaire; DERS, Difficulties in Emotion Regulation Scale; HCQ, The Health Cognitions Questionnaire; Clarity, lack of emotional clarity; Strategies, limited access to emotion regulation strategies perceived as effective; Impulse, difficulties in controlling impulsive behaviors over the distressed periods; Goals, inability to engage in goal-directed behaviors over the distressed periods; Non-acceptance, non-acceptance of negative emotions; Neuro 1, Neuro 2, Neuro 3, Parcels of NEURO; Extra 1, Extra 2, Extra 3, Parcels of EXTRA. *p < 0.05; **p < 0.01.

The initial analysis of the structural model was done based on five personality factors, mediating variables (health-related metacognition, cognitive bias, and emotion dysregulation), and cyberchondria. In this model, it was assumed that personality factors affect cyberchondria through the mediating variables in addition to having a direct effect. The results of this analysis demonstrated that personality factors (openness to experience, agreeableness, and conscientiousness) do not significantly affect other model variables (see Supplementary material for further details). Therefore, the structural model was re-performed by removing these three factors. The secondary analysis results are shown in Figure 2 and Table 3. Only the pathways of neuroticism and extraversion to cyberchondria are not significant, and the rest of the pathways demonstrated significant coefficients. As Figure 2 illustrates, neuroticism, as an exogenous variable, had a significant effect on MCQ-HA (β = 0.52, *T*-value = 8.11), DERS (β = 0.63, *T*-value = 10.17), and HCQ (β = 0.55, *T*-value = 9.52). Moreover, extraversion, as an exogenous variable, had a significant effect on MCQ-HA (β = −0.12, *T*-value = −2.09), DERS (β = −0.11, *T*-value = −2.13), and HCQ (β = −0.23, *T*-value = −4.24). Also, neuroticism (β = 0.04, *T*-value = 0.43) and extraversion (β = −0.05, *T*-value = −1.03) indicated no significant effect on the cyberchondria. On the other hand, MCQ-HA (β = 0.40, *T*-value = 7.36), DERS (β = 0.16, *T*-value = 3.01), and HCQ (β = 0.29, *T*-value = 4.79) as mediator variables revealed a significant effect on the cyberchondria. Finally, the results of fit indices demonstrate that the conceptual model of the present study is an acceptable fit with the data (χ^2^ = 924.21, χ^2^/df = 4.69, CFI = 0.92, GFI = 0.90, NFI = 0.92, IFI = 0.92, RMSEA = 0.07, SRMR = 0.06).^1^

**TABLE 3:** Direct effects between latent variables.

Independent variables	Dependent variables	B	T	SE	*p*
Neuroticism	MCQ-HA	0.52	8.11	0.064	<0.001
Neuroticism	DERS	0.63	10.17	0.062	<0.001
Neuroticism	HCQ	0.55	9.52	0.058	<0.001
Neuroticism	Cyberchondria	0.04	0.43	0.084	0.645
Extraversion	MCQ-HA	-0.12	-2.09	0.057	0.043
Extraversion	DERS	-0.11	-2.13	0.050	0.045
Extraversion	HCQ	-0.23	-4.24	0.054	0.005
Extraversion	Cyberchondria	-0.05	-1.03	0.047	0.372
MCQ-HA	Cyberchondria	0.40	7.36	0.054	<0.001
DERS	Cyberchondria	0.16	3.01	0.053	0.030
HCQ	Cyberchondria	0.29	4.79	0.060	<0.001

MCQ-HA, The meta-cognitions about health questionnaire; DERS, difficulties in emotion regulation scale; HCQ, The health cognitions questionnaire; SE, standard error.

The results demonstrated that the present model could explain 54% of cyberchondria. Furthermore, the independent variables (neuroticism and extraversion) can explain 36% of health-related metacognitions, 49% of emotional dysregulation, and 50% of health-related cognitive biases, respectively.

### 3.4. Bootstrapping

The present study utilized the Bootstrap test (iteration number = 5,000) to evaluate the indirect effects. Bootstrap provides a powerful method of assessing indirect effects (83). Evaluation of the significance of these relationships can be examined in two ways. The first method refers to significance levels, and the second examines confidence intervals. Suppose the value of zero is not placed between upper and lower limits with a 95% confidence interval (both positive or both negative). In that case, the mediation effects will be significant. The test results in Table 4 show that the neuroticism mediated by MCQ-HA, DERS, and HCQ significantly affected cyberchondria.

**TABLE 4:** Bootstrapping test indirect effect and 95% CI for the mediation model.

Independent variable	Mediator variable	Dependent variable	Standard coefficient (beta)	Standard error	95% CI		Sig
					**Lower**	**Upper**	
Neuroticism	MCQ-HA	Cyberchondria	0.208	0.059	0.115	0.309	0.001
Neuroticism	DERS	Cyberchondria	0.100	0.046	0.022	0.174	0.034
Neuroticism	HCQ	Cyberchondria	0.159	0.047	0.080	0.236	0.001
Sum of indirect			0.467	0.096	0.310	0.625	0.001
Extraversion	MCQ-HA	Cyberchondria	-0.048	0.043	-0.092	-0.006	0.047
Extraversion	DERS	Cyberchondria	-0.017	0.013	-0.040	0.015	0.466
Extraversion	HCQ	Cyberchondria	-0.066	0.030	-0.113	-0.013	0.038
Sum of indirect			-0.131	0.081	-0.252	-0.069	0.028

NEURO, MCQ-HA, The meta-cognitions about health questionnaire; DERS, difficulties in emotion regulation scale; HCQ, The health cognitions questionnaire; CI, confidence interval.

MCQ-HA (beta = 0.208, SE = 0.055, 95% CI = 0.121–0.302, *p* < 0.01), DERS (beta = 0.100, SE = 0.047, 95% CI = 0.021–0.175, *p* < 0.05), and CHQ (beta = 0.159, SE = 0.045, 95% CI = 0.084–0.232, *p* < 0.01). Furthermore, extraversion mediated by MCQ-HA and HCQ had a significant effect on cyberchondria. MCQ-HA (beta = −0.048, SE = 0.043, 95% CI = −0.096 to −0.009, *p* < 0.05) and CHQ (beta = −0.066, SE = 0.030, 95% CI = −0.112 to −0.014, *p* < 0.05). However, DERS did not play a mediating role in the relationship between extraversion and cyberchondria. DERS (beta = −0.017, SE = 0.013, 95% CI = −0.040 to 0.016, *p* > 0.05).

## 4. Discussion

The present study evaluated a prediction model of cyberchondria based on personality traits, health-related metacognition, cognitive bias, and emotion dysregulation. The study showed that neuroticism and extraversion could significantly affect cyberchondria by mediating the role of cognitive bias, health-related metacognitions, and emotion dysregulation. Also, the study’s findings indicated that the characteristics of openness to experience, agreeableness, and conscientiousness have no role in predicting cyberchondria. The results also showed that neuroticism and extraversion’s direct effect on cyberchondria was insignificant.

According to the findings derived from the structural model of the present study, the appearance of cyberchondria symptoms was rooted in personality-biological traits. Neuroticism and extroversion as personality factors in interaction with mediator variables can increase the probability of developing symptoms of cyberchondria. The findings were consistent with previous studies in confirming that neuroticism and the nature of anxiety are positively correlated with cyberchondria (39, 91, 92). On the other hand, the findings demonstrated that neuroticism was more involved in the etiology of anxiety disorders than any other personality factor (93). Individuals with high neuroticism experience extra anxiety and distress regarding ambiguous phenomena and are more focused on discovering health-related physical symptoms that could lead to cyberchondria symptoms. It is hypothesized that reducing stress and anxiety is among the ultimate purposes for which individuals with high neuroticism try to resort to online medical resources. Negative thoughts and feelings combined with decreased illness anxiety can eventually lead to cyberchondria. According to Brown et al. (94), although looking for available online information can primarily provide temporary relief from anxiety, it can secondarily lead to heightened anxiety and induced compulsive behaviors in subsequent searches for medical information.

The present study’s findings also corroborated the role of extraversion as an influential personality factor in cyberchondria and showed that extroverts experience fewer cyberchondria symptoms. Although the role of extraversion in cyberchondria has not been explicitly addressed, research have suggested extraversion interaction with neuroticism in the etiology of anxiety disorders (30, 95, 96). Additionally, in the etiology model of emotional disorders, temperament factors such as neuroticism and extraversion are considered to be general biological vulnerabilities (97, 98).

The findings indicated that the direct effect of neuroticism and extraversion factors on cyberchondria was insignificant, indicating a full mediating mechanism in the onset of cyberchondria symptoms. One of the mediating variables in the present study model was cognitive bias or health-related dysfunctional beliefs that affected cyberchondria. The findings of the current study were consistent with previous research revealing that individuals with high neuroticism consider themselves to be poorer in terms of health than individuals with low scores in this dimension, as a result of which they take the consequences of the disease more seriously (51, 99, 100). Individuals with high neuroticism believe that they are more likely to have the disease, believe in their low capacity to cope with it, and expect unpleasant consequences that cause them to experience growing anxiety and distress. One way of addressing these concerns is to check online and search for medical information.

Unlike individuals with high neuroticism, extroverts report fewer physical symptoms and negative experiences regarding their health (52). People with high extroversion are more likely to engage in exercise and eat healthy foods and vitamins, affecting their health (52). Optimistic beliefs influence these behaviors in extroverts about health that have greater self-efficacy in dealing with disease and expecting further positive outcomes (101, 102). This could elucidate the negative effect of extraversion on dysfunctional beliefs and the indirect negative impact on cyberchondria. Once extroverts hold positive beliefs and a sort of optimism about their health, they will be less inclined to search for medical information online. However, there is a possibility for extroverts to be influenced by unrealistic optimism (103, 104). They might underestimate the risk of the disease and feel less vulnerable to the negative consequences of the disease in the future.

Another mechanism for the emergence of cyberchondria symptoms in the structural model of the current study was the mediating role of health-related metacognitions. Studies demonstrated that neuroticism served as a natural basis for activating maladaptive metacognitions that led to or exacerbated psychological distress (60, 105). Metacognitive beliefs (e.g., beliefs about one’s thoughts and coping strategies) can mediate the relationship between neuroticism and psychological distress (61, 62, 105). Given that psychological distress is among the central components of cyberchondria, negative metacognitive beliefs can lead to more anxiety, and the emergence of cyberchondria symptoms becomes a defective cycle to reduce anxiety.

On the other hand, negative metacognitive beliefs (e.g., the uncontrollable nature of the disease-related thoughts) are associated with cyberchondria (39, 106) and health anxiety (59). Individuals with high neuroticism cannot regulate their beliefs and thoughts regarding the disease, and controlling them is a tough challenge (58). People with negative metacognitive beliefs (for instance, one who believes their thoughts about the symptoms of the disease are uncontrollable) may seek out the disease information to reduce the uncertainty and inadequacy of their data (22). Throughout the process, there is a possibility that the individual is confronted with threatening information or engaged in numerous reassurances being raised from information seeking online. These negative metacognitive beliefs not only can evoke the symptoms of cyberchondria on their own, but they can also predict cognitive bias toward health-related information (55). These cognitive biases are strongly associated with addictive behaviors (107) that are abundantly being witnessed in cyberchondria which involves frequent and extreme online medical searches (14).

Emotional dysregulation was another variable that significantly mediated the effects of neuroticism and extraversion on cyberchondria. Numerous studies evaluated the impact of factors of neuroticism and extraversion on emotion regulation strategies. The findings of these studies revealed that neuroticism had a positive relationship with difficulty in emotion regulation (108, 109), rumination, and suppression (110). The studies also demonstrated a negative relationship between neuroticism and reappraisal strategy (111, 112) and management and regulation of impulses (113, 114). Conversely, extroversion has demonstrated a positive correlation with problem-solving strategy and seeking support (115), using repair (116), and positive reinterpretation (117). They also showed the existence of a negative correlation between extroversion and suppression (111, 118, 119), alexithymia (120), and avoidance (121). The role of emotion dysregulation in the development of health anxiety has also been studied (66, 69).

In the context of health anxiety, emotion dysregulation in the form of an inability to recognize and understand emotional experiences causes individuals to interpret bodily emotions and symptoms as medical problems (66). This self-regulation can increase emotional arousal and lead to misinterpretation of emotional and physical arousal and health anxiety (69). The emotional distress resulting from focusing on bodily emotions, misunderstanding, and attempting to control them leads to extreme reassurance that directs people to search online for medical resources and cyberchondria.

Overall, the present study’s findings supported the idea that neuroticism and extraversion personality traits are the two personality factors underlying the development of cyberchondria. High neuroticism and low extraversion can be considered cyberchondria personality-biological vulnerability factors. However, the findings of the present analysis also demonstrated that the effect, as mentioned earlier, is not direct, and mediating mechanisms are involved. These mediating mechanisms included the three main domains of dysfunctional health-related beliefs, meta-cognitions about health, and emotion dysregulation, either of which transmitted the effects of personality factors to cyberchondria in the designated manner.

The present study’s findings can be notable in the field of clinical application. Cyberchondria is not considered a distinct diagnostic disorder, and there is a high probability that individuals with this phenomenon will not be noticed and ignored. The present study’s findings revealed that cyberchondria is a phenomenon rooted in personality factors and emerges with cognitive, metacognitive, and emotional mediating mechanisms. Understanding the main factors affecting the formation and persistence of cyberchondria, in addition to increasing our awareness of this emerging phenomenon, can be a basis for the development of prevention and treatment approaches, and thus, both reduce the produced suffering and lead to a reduction in the economic costs of the problem.

### 4.1. Limitations

It is worth mentioning that the limitations of the present study should be considered prior to any generalization of the findings. First, the present research’s cross-sectional design and correlational nature precluded conclusions regarding causal relationships. Longitudinal studies or controlled experiments are recommended to comprehensively determine a causal relationship between personality traits and cognitive, metacognitive, and emotional factors with cyberchondria. Second, the utilized self-report evaluation method to collect data from the present study is prone to social desirability and may cause inflated correlations between research variables. Third, it should be borne in mind that the present study sample consisted mainly of highly educated individuals, which challenges the generalizability of its findings to other groups. Therefore, our findings require further analysis in more diverse samples. Finally, the methodological limitation of the current research is the problem of Multiplicity. Multiplicity refers to the situation in which the type 1 error is inflated due to multiple testing. In the SEM framework, when the number of parameters changes due to the modifications of the model, the problem of Multiplicity affects the type error 1 and the significance of the relationships. Despite these limitations, the current study was a remarkable pace toward enhancing the present knowledge of the nature of cyberchondria.

## Data availability statement

The raw data supporting the conclusions of this article will be made available by the authors, without undue reservation.

## Ethics statement

Ethical review and approval was not required for the study on human participants in accordance with the local legislation and institutional requirements. All participants provided their written informed consent to participate in this study.

## Author contributions

MN gathered data, performed the statistical analysis, and prepared the manuscript. MN, SM, and MA designed the study. SM, MA, and MMA reviewed and revised the manuscript. All authors interpreted the data and approved the final version of the manuscript.

## Footnotes

1 Although based on the mentioned references, the fit indices for the structural model are within the acceptable range, several researchers considered a stricter cutoff for these indices (RMSEA ≤ 0.06, GFI, CFI, NFI, and IFI ≥ 0.95) (81, 89, 90). Nevertheless, this may not be too problematic given that the fit index is only away from the cutoff by a small amount.

## Supplementary material

Supplementary Figure 1 Initial model with standardized coefficients.

Supplementary Figure 2 Initial model with *T*-value.

Supplementary Table 1 Full output of LISREL.
