# Supplementary figures and images for: The structural model of cyberchondria based on personality traits, health-related metacognition, cognitive bias, and emotion dysregulation
Source: Front Psychiatry. 2023 Jan 9;13:960055. doi: 10.3389/fpsyt.2022.960055 (PMC9869141; doi:10.3389/fpsyt.2022.960055)

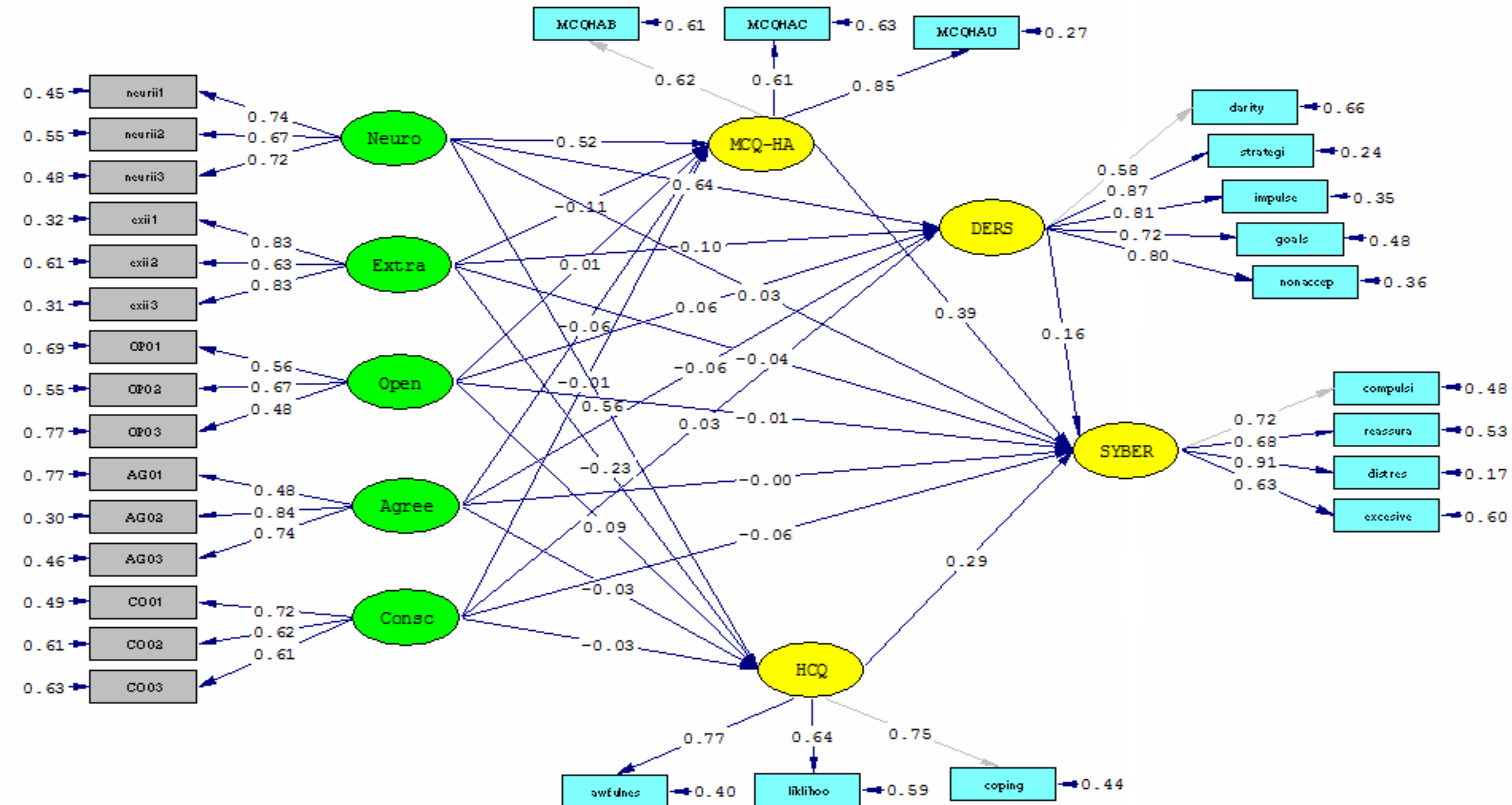

Chi-Square=1973.88, df=372, P-value=0.00000, RMSEA=0.078

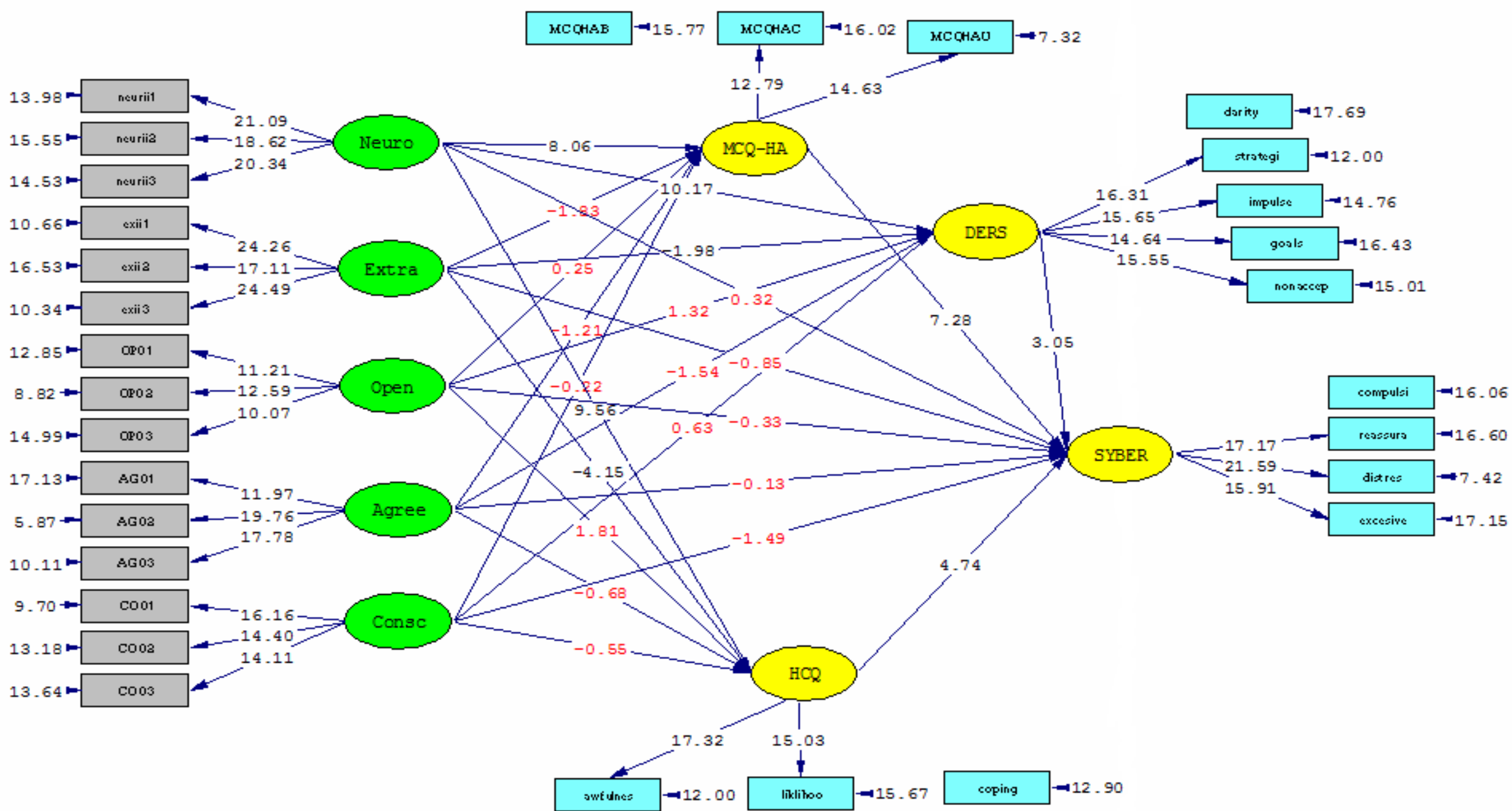

Chi-Square=1973.88, df=372, P-value=0.00000, RMSEA=0.078

Supplement: Supplementary Figure 1 — Initial model with standardized coefficients. [file Data_Sheet_1.PDF]
