# Supplementary material for: The structural model of cyberchondria based on personality traits, health-related metacognition, cognitive bias, and emotion dysregulation
Source: Front Psychiatry. 2023 Jan 9;13:960055. doi: 10.3389/fpsyt.2022.960055 (PMC9869141; doi:10.3389/fpsyt.2022.960055)
Supplement: Supplementary Table 1 — Full output of LISREL. [file Data_Sheet_2.PDF]

DATE: 11/13/2022
TIME: 20:16

L I S R E L 8.80

BY

Karl G. Jöreskog and Dag Sörbom

This program is published exclusively by
Scientific Software International, Inc.
7383 N. Lincoln Avenue, Suite 100
Lincolnwood, IL 60712, U.S.A.
Phone: (800)247-6113, (847)675-0720, Fax: (847)675-2140
Copyright by Scientific Software International, Inc., 1981-2006
Use of this program is subject to the terms specified in the
Universal Copyright Convention.
Website: www.ssicentral.com

The following lines were read from file
E:\data\SPSS\study1\DATAKOL\lisreel\PRIMERY\yhdd.SPJ:

```
Latent Variables  'MCQ-HA' DERS HCQ SYBER Neuro Extra Open Agree Consc
Relationships
MCQHAB = 'MCQ-HA'
MCQHAC = 'MCQ-HA'
MCQHAU = 'MCQ-HA'
clarity = DERS
strategi = DERS
impulse = DERS
goals = DERS
nonaccep = DERS
coping = HCQ
liklihoo = HCQ
awfulnes = HCQ
compulsi = SYBER
reassura = SYBER
distres = SYBER
excesive = SYBER
neurii1 = Neuro
neurii2 = Neuro
neurii3 = Neuro
exii1 = Extra
exii2 = Extra
exii3 = Extra
OP01 = Open
OP02 = Open
OP03 = Open
AG01 = Agree
AG02 = Agree
AG03 = Agree
CO01 = Consc
CO02 = Consc
CO03 = Consc
SYBER = 'MCQ-HA' DERS HCQ
'MCQ-HA' = Neuro Extra Open Agree Consc
DERS = Neuro Extra Open Agree Consc
```

```
HCQ = Neuro Extra Open Agree Consc
SYBER = Neuro Extra Open Agree Consc
Path Diagram
Admissibility Check = Off
Iterations = 1000
End of Problem
```

Covariance Matrix

	MCQHAB	MCQHAC	MCQHAU	clarity	strategi	impulse
MCQHAB	5.05					
MCQHAC	3.43	9.63				
MCQHAU	2.78	3.82	5.97			
clarity	0.96	1.21	1.00	1.88		
strategi	2.73	3.90	5.10	2.62	15.99	
impulse	1.58	2.59	3.47	1.77	7.07	6.99
goals	1.31	2.37	2.52	1.42	6.45	4.64
nonaccep	2.03	3.12	3.20	1.72	8.46	4.54
coping	1.23	2.95	4.72	1.44	7.00	3.65
liklihoo	0.63	1.68	1.94	0.60	4.40	2.01
awfulnes	1.21	2.25	3.51	0.70	5.32	2.51
compulsi	3.57	1.83	4.63	2.50	6.23	5.42
reassura	3.51	2.60	4.17	1.24	4.97	3.75
distres	4.83	6.14	9.33	3.45	11.75	9.21
excesive	4.40	2.87	4.80	2.28	5.16	4.88
neurii1	0.86	1.54	3.00	1.48	5.99	3.56
neurii2	1.15	2.44	2.34	1.45	4.75	3.49
neurii3	1.55	2.89	2.48	2.09	5.78	3.15
exii1	-2.01	-3.00	-2.77	-1.65	-5.31	-3.57
exii2	-2.46	-1.78	-2.01	-1.11	-2.67	-1.92
exii3	-1.43	-1.81	-2.30	-1.36	-4.65	-3.19
OP01	-0.70	-1.30	-0.56	-0.39	-0.03	-0.64
OP02	-0.29	-0.44	-0.35	-0.31	-0.79	-0.77
OP03	-0.46	-0.92	-0.82	-0.18	-0.69	-0.47
AG01	-0.02	-0.37	-0.59	-0.47	-0.96	-1.01
AG02	-0.45	-0.65	-0.76	-0.44	-1.08	-1.20
AG03	-0.72	-0.58	-1.19	-0.28	-1.25	-0.98
CO01	-0.18	-0.44	-0.15	-0.21	-0.04	-0.39
CO02	-0.37	-0.86	-0.83	-0.28	-1.05	-0.62
CO03	-0.64	-0.41	-0.45	-0.01	-0.42	-0.49

Covariance Matrix

	goals	nonaccep	coping	liklihoo	awfulnes	compulsi
goals	6.81					
nonaccep	3.85	7.89				
coping	3.42	3.85	25.22			
liklihoo	2.50	2.71	7.47	8.89		
awfulnes	2.39	2.75	8.93	4.68	9.87	
compulsi	2.27	5.03	8.71	4.30	5.03	24.95
reassura	2.48	3.68	3.07	2.63	3.40	10.67
distres	6.17	7.83	15.76	7.40	10.50	21.72
excesive	1.97	4.13	2.31	1.00	3.09	13.49
neurii1	2.44	3.11	7.16	2.39	3.96	4.06
neurii2	3.11	2.61	4.49	2.16	3.15	2.91
neurii3	2.82	3.57	7.18	3.19	4.81	7.38
exii1	-2.91	-2.99	-6.34	-3.37	-4.49	-5.84
exii2	-2.47	-2.11	-3.85	-1.38	-2.16	-5.51

exii3	-3.37	-2.20	-6.55	-3.27	-4.20	-6.20
OP01	-0.27	0.02	0.06	-0.19	-0.29	-0.65
OP02	-0.99	-0.25	-0.82	-0.62	-0.66	-0.88
OP03	-0.28	-0.32	-0.26	-0.65	-0.49	-0.85
AG01	-0.94	-0.52	-1.51	-1.15	-1.33	-2.20
AG02	-1.21	-0.76	-0.81	-1.29	-0.85	-2.42
AG03	-0.74	-1.29	-1.62	-1.41	-0.82	-2.25
CO01	-0.41	0.05	-0.57	-0.47	-0.21	-1.23
CO02	-0.52	-0.34	-1.62	-0.87	-0.95	-1.30
CO03	-0.26	-0.06	-0.89	-0.33	-0.59	-2.05

Covariance Matrix (continued)

	reassura	distres	excesive	neurii1	neurii2	neurii3
reassura	19.54					
distres	17.36	42.18				
excesive	17.55	19.83	31.22			
neurii1	2.69	8.57	2.74	10.42		
neurii2	2.86	6.95	2.85	5.42	10.14	
neurii3	4.75	11.00	4.05	7.38	6.73	15.84
exii1	-3.38	-9.02	-2.55	-3.67	-4.55	-6.56
exii2	-3.85	-6.35	-5.78	-2.07	-2.47	-4.58
exii3	-3.01	-8.34	-2.41	-3.91	-3.47	-6.02
OP01	-1.06	-1.26	-2.22	-0.83	-1.31	-0.98
OP02	-0.67	-1.83	-0.52	-1.38	-0.77	-1.40
OP03	-1.08	-1.64	-0.70	-0.24	-0.49	-1.18
AG01	-1.15	-2.64	-1.21	-0.67	-0.95	-2.02
AG02	-0.52	-2.61	-0.98	-0.74	-0.97	-1.20
AG03	-0.68	-2.75	-0.85	-0.80	-0.84	-1.31
CO01	-0.09	-1.43	0.06	-0.20	-0.18	-0.90
CO02	-0.97	-2.67	-2.28	-0.95	-1.31	-1.16
CO03	-1.52	-2.16	-1.70	-0.63	-0.68	-0.62

Covariance Matrix (continued)

	exii1	exii2	exii3	OP01	OP02	OP03
exii1	15.07					
exii2	6.67	11.35				
exii3	9.59	6.45	12.94			
OP01	1.49	0.62	0.45	10.30		
OP02	1.68	1.39	1.30	3.30	7.92	
OP03	1.00	0.83	0.11	2.30	2.34	6.64
AG01	1.69	1.00	1.61	1.07	1.08	0.75
AG02	1.87	1.17	1.77	-0.02	0.47	0.76
AG03	2.17	1.22	1.69	0.16	0.03	1.64
CO01	1.29	0.73	0.70	0.58	0.93	0.52
CO02	1.09	0.58	0.86	1.90	1.42	0.67
CO03	0.51	0.31	0.63	0.65	0.82	-0.09

Covariance Matrix (continued)

	AG01	AG02	AG03	CO01	CO02	CO03

**AG01**	9.47					
**AG02**	4.53	12.83				
**AG03**	3.29	7.39	11.13			
**CO01**	1.05	2.62	2.94	9.81		
**CO02**	1.87	0.48	0.82	4.59	11.48	
**CO03**	1.14	0.85	0.10	4.15	4.13	9.36

Number of Iterations = 74

LISREL Estimates (Maximum Likelihood)

Measurement Equations

```
MCQHAB = 1.40*MCQ-HA, Errorvar.= 3.10 , R² = 0.39
(0.20)
15.77

MCQHAC = 1.88*MCQ-HA, Errorvar.= 6.08 , R² = 0.37
(0.15)                  (0.38)
12.79                   16.02

MCQHAU = 2.08*MCQ-HA, Errorvar.= 1.64 , R² = 0.73
(0.14)                  (0.22)
14.63                   7.32

clarity = 0.79*DERS, Errorvar.= 1.25  , R² = 0.34
(0.071)
17.69

strategi = 3.49*DERS, Errorvar.= 3.84 , R² = 0.76
(0.21)                (0.32)
16.31                 12.00

impulse = 2.13*DERS, Errorvar.= 2.45 , R² = 0.65
(0.14)                (0.17)
15.65                 14.76

goals = 1.89*DERS, Errorvar.= 3.25 , R² = 0.52
(0.13)                (0.20)
14.64                 16.43

nonaccep = 2.24*DERS, Errorvar.= 2.87 , R² = 0.64
(0.14)                (0.19)
15.55                 15.01

coping = 3.77*HCQ, Errorvar.= 11.04, R² = 0.56
(0.86)
12.90

liklihoo = 1.91*HCQ, Errorvar.= 5.25 , R² = 0.41
(0.13)               (0.34)
15.03                15.67

awfulnes = 2.43*HCQ, Errorvar.= 3.96 , R² = 0.60
(0.14)               (0.33)
17.32                12.00

compulsi = 3.56*SYBER, Errorvar.= 11.84, R² = 0.52
```

```
(0.74)
16.06

reassura = 3.00*SYBER, Errorvar.= 10.24, R² = 0.47
(0.17)                 (0.62)
17.17                  16.60

distres = 5.85*SYBER, Errorvar.= 6.85 , R² = 0.83
(0.27)                 (0.92)
21.59                  7.42

excesive = 3.52*SYBER, Errorvar.= 18.44, R² = 0.40
(0.22)                 (1.08)
15.91                  17.15


neurii1 = 2.40*Neuro, Errorvar.= 4.68 , R² = 0.55
(0.11)                 (0.33)
21.09                  13.98

neurii2 = 2.15*Neuro, Errorvar.= 5.53 , R² = 0.45
(0.12)                 (0.36)
18.62                  15.55

neurii3 = 2.87*Neuro, Errorvar.= 7.58 , R² = 0.52
(0.14)                 (0.52)
20.34                  14.53

exii1 = 3.21*Extra, Errorvar.= 4.79 , R² = 0.68
(0.13)                 (0.45)
24.26                  10.66

exii2 = 2.11*Extra, Errorvar.= 6.91 , R² = 0.39
(0.12)                 (0.42)
17.11                  16.53

exii3 = 2.99*Extra, Errorvar.= 3.99 , R² = 0.69
(0.12)                 (0.39)
24.49                  10.34

OP01 = 1.78*Open, Errorvar.= 7.13 , R² = 0.31
(0.16)                (0.55)
11.21                 12.85

OP02 = 1.88*Open, Errorvar.= 4.38 , R² = 0.45
(0.15)                (0.50)
12.59                 8.82

OP03 = 1.24*Open, Errorvar.= 5.11 , R² = 0.23
(0.12)                (0.34)
10.07                 14.99

AG01 = 1.48*Agree, Errorvar.= 7.28 , R² = 0.23
(0.12)                 (0.43)
11.97                  17.13

AG02 = 2.99*Agree, Errorvar.= 3.88 , R² = 0.70
(0.15)                 (0.66)
19.76                  5.87

AG03 = 2.45*Agree, Errorvar.= 5.10 , R² = 0.54
(0.14)                 (0.50)
17.78                  10.11

CO01 = 2.24*Consc, Errorvar.= 4.79 , R² = 0.51
(0.14)                 (0.49)
16.16                  9.70
```

```
CO02 = 2.10*Consc, Errorvar.= 7.05 , R² = 0.39
(0.15)                  (0.54)
14.40                   13.18

CO03 = 1.86*Consc, Errorvar.= 5.92 , R² = 0.37
(0.13)                  (0.43)
14.11                   13.64
```

Structural Equations

```
MCQ-HA = 0.52*Neuro - 0.11*Extra + 0.013*Open - 0.056*Agree - 0.011*Consc,
Errorvar.= 0.64  , R² = 0.36
(0.065)      (0.058)      (0.052)      (0.046)       (0.050)
(0.082)
8.06        -1.83         0.25        -1.21         -0.22                   7.78

DERS = 0.64*Neuro - 0.10*Extra + 0.061*Open - 0.062*Agree + 0.028*Consc, Errorvar.=
0.51  , R² = 0.49
(0.063)      (0.051)      (0.046)      (0.041)       (0.044)
(0.068)
10.17       -1.98         1.32        -1.54          0.63                   7.45

HCQ = 0.56*Neuro - 0.23*Extra + 0.091*Open - 0.030*Agree - 0.026*Consc, Errorvar.=
0.49  , R² = 0.51
(0.059)      (0.055)      (0.050)      (0.044)       (0.047)
(0.058)
9.56        -4.15         1.81        -0.68         -0.55                   8.51

SYBER = 0.39*MCQ-HA + 0.16*DERS + 0.29*HCQ + 0.028*Neuro - 0.041*Extra - 0.015*Open
- 0.0048*Agree - 0.061*Consc,
(0.054)       (0.053)      (0.061)    (0.087)       (0.048)       (0.044)
(0.038)        (0.041)
7.28          3.05         4.74       0.32          -0.85         -0.33         -0.13
        -1.49

Errorvar.= 0.45  , R² = 0.55
(0.048)
9.40
```

Reduced Form Equations

	**= 0.52*Neu**	**ro - 0.11*E**	**xtra + 0.01**	**3*Open - 0.**	**056*Agree -**	**0.011*Cons**	**c, Errorvar**	**.= 0**
	8.06 -1.83		0.25	-1.	21	-0.22		
**DERS**		=	0.64*Neuro	-	0.10*Extra	+	0.061*Open	
		(0.063)	(0.051)	(0.046)	(0.041)	(0.044)		
		10.17	-1.98	1.32	-1.54	0.63		
**HCQ**		=	0.56*Neuro	-	0.23*Extra	+	0.091*Open	
		(0.059)	(0.055)	(0.050)	(0.044)	(0.047)		
		9.56	-4.15	1.81	-0.68	-0.55		
**SYBER**		=	0.50*Neuro	-	0.17*Extra	+	0.027*Open	
		(0.058)	(0.053)	(0.048)	(0.042)	(0.046)		
		8.61	-3.10	0.55	-1.08	-1.49		

Correlation Matrix of Independent Variables

	**Neuro**	**Extra**	**Open**	**Agree**	**Consc**
**Neuro**	1.00				

Extra	-0.59	1.00			
	(0.03)				
	-16.89				
Open	-0.25	0.22	1.00		
	(0.05)	(0.05)			
	-4.60	4.26			
Agree	-0.15	0.22	0.12	1.00	
	(0.05)	(0.04)	(0.05)		
	-3.12	5.02	2.21		
Consc	-0.13	0.13	0.24	0.27	1.00
	(0.05)	(0.05)	(0.06)	(0.05)	
	-2.53	2.69	4.24	5.65	

Covariance Matrix of Latent Variables

	MCQ-HA	DERS	HCQ	SYBER	Neuro	Extra
MCQ-HA	1.00					
DERS	0.42	1.00				
HCQ	0.42	0.49	1.00			
SYBER	0.63	0.52	0.58	1.00		
Neuro	0.59	0.69	0.68	0.60	1.00	
Extra	-0.43	-0.48	-0.55	-0.47	-0.59	1.00
Open	-0.15	-0.12	-0.11	-0.15	-0.25	0.22
Agree	-0.16	-0.17	-0.16	-0.17	-0.15	0.22
Consc	-0.11	-0.07	-0.12	-0.16	-0.13	0.13

Covariance Matrix of Latent Variables (continued)

	Open	Agree	Consc
Open	1.00		
Agree	0.12	1.00	
Consc	0.24	0.27	1.00

Goodness of Fit Statistics

Degrees of Freedom = 372
Minimum Fit Function Chi-Square = 1948.45 (P = 0.0)
Normal Theory Weighted Least Squares Chi-Square = 1973.88 (P = 0.0)
Estimated Non-centrality Parameter (NCP) = 1601.88
90 Percent Confidence Interval for NCP = (1466.46 ; 1744.75)

Minimum Fit Function Value = 2.78
Population Discrepancy Function Value (F0) = 2.28
90 Percent Confidence Interval for F0 = (2.09 ; 2.49)
Root Mean Square Error of Approximation (RMSEA) = 0.078
90 Percent Confidence Interval for RMSEA = (0.075 ; 0.082)
P-Value for Test of Close Fit (RMSEA < 0.05) = 0.00

Expected Cross-Validation Index (ECVI) = 3.08
90 Percent Confidence Interval for ECVI = (2.88 ; 3.28)
ECVI for Saturated Model = 1.32
ECVI for Independence Model = 29.58

Chi-Square for Independence Model with 435 Degrees of Freedom = 20703.70
Independence AIC = 20763.70
Model AIC = 2159.88

```
Saturated AIC = 930.00
Independence CAIC = 20930.37
Model CAIC = 2676.52
Saturated CAIC = 3513.24

Normed Fit Index (NFI) = 0.91
Non-Normed Fit Index (NNFI) = 0.91
Parsimony Normed Fit Index (PNFI) = 0.77
Comparative Fit Index (CFI) = 0.92
Incremental Fit Index (IFI) = 0.92
Relative Fit Index (RFI) = 0.89

Critical N (CN) = 158.95


Root Mean Square Residual (RMR) = 0.83
Standardized RMR = 0.060
Goodness of Fit Index (GFI) = 0.84
Adjusted Goodness of Fit Index (AGFI) = 0.80
Parsimony Goodness of Fit Index (PGFI) = 0.67
```

The Modification Indices Suggest to Add the

Path to	from	Decrease in Chi-Square	New Estimate
MCQHAB	HCQ	23.9	-0.48
MCQHAC	SYBER	23.9	-0.83
MCQHAU	DERS	57.4	0.75
MCQHAU	HCQ	59.3	0.84
MCQHAU	SYBER	68.5	1.39
clarity	SYBER	11.6	0.19
strategi	HCQ	11.3	0.43
impulse	MCQ-HA	17.5	0.35
impulse	SYBER	33.2	0.50
awfulnes	MCQ-HA	19.7	0.55
compulsi	MCQ-HA	9.2	-0.70
reassura	HCQ	45.1	-1.32
distres	MCQ-HA	12.9	1.06
distres	DERS	16.6	0.95
distres	HCQ	116.9	2.99
excesive	DERS	8.0	-0.62
excesive	HCQ	88.1	-2.43
neurii1	Extra	18.2	0.70
neurii3	Extra	21.3	-0.92
exii3	Open	9.0	-0.41
OP03	Agree	8.6	0.32
AG01	Open	12.8	0.49
CO01	Agree	30.2	0.77
CO02	Open	8.2	0.46
CO02	Agree	8.8	-0.43
CO03	Agree	9.7	-0.40
MCQ-HA	DERS	109.4	0.67
MCQ-HA	HCQ	16.8	0.29
MCQ-HA	SYBER	94.4	2.20
DERS	MCQ-HA	109.4	0.53
DERS	SYBER	109.9	1.24
HCQ	MCQ-HA	16.8	0.23
HCQ	SYBER	22.0	0.64

The Modification Indices Suggest to Add an Error Covariance

Between	and	Decrease in Chi-Square	New Estimate
DERS	MCQ-HA	109.4	0.34
HCQ	MCQ-HA	16.8	0.14
MCQHAC	MCQHAB	40.7	1.37
MCQHAU	MCQHAB	16.9	-0.96
clarity	MCQHAB	17.4	0.34
clarity	MCQHAU	27.6	-0.40
strategi	MCQHAU	15.3	0.62
impulse	MCQHAB	9.0	-0.37
impulse	MCQHAU	17.4	0.48
impulse	strategi	31.3	-1.17
goals	impulse	55.8	1.03
nonaccep	MCQHAC	9.5	0.57
nonaccep	strategi	84.0	2.02
nonaccep	impulse	10.9	-0.47
nonaccep	goals	17.2	-0.61
coping	MCQHAB	7.9	-0.76
liklihoo	goals	9.0	0.53
awfulnes	MCQHAU	23.0	0.75
awfulnes	clarity	15.7	-0.40
awfulnes	strategi	18.7	0.90
compulsi	MCQHAB	9.8	0.81
compulsi	MCQHAC	21.3	-1.66
compulsi	clarity	15.2	0.62
compulsi	goals	26.7	-1.36
compulsi	awfulnes	9.1	-0.98
reassura	MCQHAB	20.2	1.07
reassura	coping	33.7	-2.81
distres	MCQHAB	36.4	-1.65
distres	MCQHAU	30.4	1.53
distres	impulse	8.1	0.71
distres	coping	24.2	2.77
distres	awfulnes	9.1	1.05
distres	reassura	29.1	-3.81
excesive	MCQHAB	26.1	1.61
excesive	clarity	10.6	0.63
excesive	goals	8.7	-0.95
excesive	coping	22.7	-3.05
excesive	liklihoo	12.0	-1.43
excesive	reassura	217.0	8.78
excesive	distres	46.1	-5.84
neurii1	MCQHAB	18.0	-0.73
neurii1	MCQHAC	19.8	-1.07
neurii1	MCQHAU	12.5	0.59
neurii1	strategi	8.9	0.66
neurii1	goals	14.7	-0.67
neurii1	coping	21.8	1.66
neurii1	liklihoo	9.8	-0.71
neurii2	strategi	8.0	-0.64
neurii2	impulse	8.4	0.48
neurii2	goals	10.8	0.60
neurii2	nonaccep	10.8	-0.59

neurii2	compulsi	10.7	-1.14
neurii3	MCQHAU	54.3	-1.55
neurii3	clarity	21.3	0.61
neurii3	impulse	20.3	-0.90
neurii3	compulsi	18.4	1.81
neurii3	neurii1	11.8	1.23
neurii3	neurii2	10.2	1.09
exii1	MCQHAC	7.9	-0.74
exii1	goals	10.0	0.61
exii1	excesive	8.0	1.26
exii1	neurii1	9.4	0.76
exii1	neurii2	9.7	-0.80
exii2	MCQHAB	37.5	-1.20
exii2	strategi	12.7	0.88
exii2	reassura	9.4	-1.08
exii2	distres	20.5	1.79
exii2	excesive	52.1	-3.35
exii3	goals	16.0	-0.71
exii3	nonaccep	20.4	0.78
OP01	excesive	12.2	-1.72
OP02	goals	8.7	-0.53
OP03	neurii1	7.9	0.62
OP03	exii3	10.6	-0.73
AG03	nonaccep	11.0	-0.61
AG03	OP02	10.5	-0.81
AG03	OP03	25.0	1.18
AG03	AG01	11.3	-1.73
AG03	AG02	9.8	5.17
CO01	AG01	12.6	-0.99
CO01	AG03	27.5	1.39
CO02	OP01	8.9	0.98
CO02	AG01	16.7	1.27
CO02	AG02	14.4	-1.17
CO03	AG03	19.7	-1.18

Time used: 0.297 Seconds
